# Applicability of computed tomography preoperative assessment of the LAA in LV summit ablations

**DOI:** 10.1007/s10840-020-00817-8

**Published:** 2020-07-14

**Authors:** Marcin Kuniewicz, M. Krupiński, M. Gosnell, K. Budnicka, N. Jakob, G. Karkowski, M. Urbańczyk-Zawadzka, J. Lelakowski, J. Walocha

**Affiliations:** 1 Department of Anatomy, Jagiellonian University Medical College, Krakow, Poland; 2 Department of Electrocardiology, Institute of Cardiology, John Paul II Hospital, Jagiellonian University Medical College, Krakow, Poland; 3 Department of Radiology and Diagnostic Imaging, John Paul II Hospital, Krakow, Poland

**Keywords:** Left ventricular summit, Left atrial appendage, Catheter ablation

## Abstract

**Purpose:**

Ventricular arrhythmias originating from the left ventricular summit (LVS) may present with challenges for catheter ablation. Recently, the left atrial appendage (LAA) became a new vantage point for mapping and ablating arrhythmias from that region, but data of possible usefulness is limited.

**Methods:**

From September to December 2019, we retrospectively analyzed 48 consecutive patient hearts (20 male; mean age 57.9y ± 11.56) undergoing diagnostic coronary vessel imaging in 64 dual-source computer tomography angiography (CTA). Distances from the LAA to the LVS, LAA shape type, and coronary arteries in the LVS region were measured. Also, we compared the true LVS area from CTA with a calculated formula derived from LVS definition.

**Results:**

The mean LVS area calculated from the formula was 291.58 mm^2^ (± 115.5) while the true area calculated from CT was 263.33 mm^2^ (± 99.49) (*p* = 0.44). The mean inaccessible area was 133.42 mm^2^ (± 72.89), accessible 95.67 mm^2^ (± 72.77). The mean LAA coverage over LVS was 196.08 mm^2^—which is approximately 75% of LVS size in general. The most common LAA shape was chicken wing (50%); windsock has the highest accessible area coverage on average (80.23%), followed by chicken wing (59.88%), broccoli (47.72%), and cactus (46.98%). The mean distance from LAA to the surface was 5.14 mm (1.5 to 10 mm) and was not correlated with BMI. LAA has a 98% coverage over the point of transition between the great cardiac vein and anterior interventricular vein.

**Conclusion:**

Angio-CT assessment of the LAA over the LVS structures may be helpful in decision making before an ablation procedure. LAA appears to be a promising mapping approach in LVS arrhythmias.

## Introduction

The left ventricular summit (LVS) is an epicardial region surrounded by the bifurcation of the left coronary artery (LCA), left anterior descending artery (LAD), and circumflex artery (Cx), termed by McAlpine in 1975 [1]. The most inferior boundary of this region is delineated by an arched line, with the radius of this arc as the distance from the bifurcation of the LCA to the first septal perforator (SP) [2]. Inside this trigon, the great cardiac vein (GCV) transitions from the left atrioventricular groove towards the anterior atrioventricular groove as the anterior interventricular cardiac vein (AIVV). This transitional region of the GCV into the AIVV is a significant source of epicardial idiopathic ventricular arrhythmias (VA) [3]. The GCV/AIVV bisects the LVS into a superior portion that is in close proximity to the proximal coronary arteries and overlying epicardial fat (inaccessible area) and an inferior portion that may be accessible to epicardial catheter ablation (accessible area) [2]. Successful catheter ablation from the LVS may require various approaches as it presents the challenge to be in close enough proximity to target the arrhythmia source. The first successful RF ablation from the LAA was performed in 2002 [4], while in 2018, the left atrial appendage was described as a vantage point for mapping and ablating VA originating from the LVS region in two case reports [5, 6]. Inspired by this approach, the aim of this study was the evaluation of the accessibility of LAA over the LVS in computer tomography angiography (CTA). Various studies are used to image the size and shape of the LAA; however, they do not depict the LVS coverage, nor the distance to the epicardium of the LVS. The second issue was to compare the real size of the LVS image derived from CTA to the equation proposed by Yamada.

## Methods

From September to December 2019, we retrospectively analyzed 48 consecutive patient hearts (20 males; mean age 57.9 years ± 11.56). These hearts underwent diagnostic coronary vessel imaging with a 64 dual-source computer tomography angiography (CTA), measuring the size of the LVS from the first SP and LAA coverage over the LVS. The area was measured in all of the patients at the same angle using a dedicated workstation. Measurements were calculated on a rendered image in SketchAndCalc software after distance calibration was derived from the CTA workstation. Distances from the LAA to the LVS, LAA shape type, and coronary arteries/branches in the LVS region were measured. Also, we compared the true LVS area with a calculated formula derived from the LVS definition. We can assume that the area of the LVS is a circular sector with a radius measured from the LCA bifurcation to the first dominant SP [2]. The central angle for this formula is the angle between the Cx and LAD. Having only two measurements, we should be able to estimate the size of the LVS after obtaining the coronary angiography (CA) results using Yamada’s formula. Depicting the GCV, we also assessed the size of the accessible and inaccessible area. After measuring the size of the LVS, we looked for the presence of any incidental arterial vessels arising from the LAD—diagonal 1 (Dx1) or marginal 1 (Mg1) trespassing inside the LVS. These measurements were to assess the usage of the LAA and possible coverage over the accessible and inaccessible area and the distance between the LAA and myocardium of left ventricle (LV). Finally, the aim of the study was to correlate the shape of the LAA to the coverage range over the LVS.

### Cardiac CT protocol

The CTA examinations were performed using dual-source CT (Somatom Definition, Siemens, Erlangen, Germany). The contrast-enhanced acquisitions were performed during inspiratory breath hold with the collimation of 0.75 mm. An iodinated contrast agent was injected with an injection rate of 5 ml/s [7]. Images were reconstructed with an image matrix of 512 × 512 pixels. The postprocessing and study evaluation were performed using a dedicated workstation (Syngovia, Siemens, Erlangen, Germany). Analyses of vein flow over LV were done by volume rendered reconstructions, sagittal, coronal and transverse presentations. Rendered reconstructions were used to identify the LVS, left coronary arteries branches, its divisions, LAA, GCV/ACV in LVS region, distances and angles between branches of LCA.

### Statistical analysis

The results were calculated as mean values and corresponding standard deviations. A chi-square test was used to evaluate difference in dichotomous data. A *P* value < 0.05 was considered significant. The *t* test was used to evaluate differences in diameters, angles, and volumes between study control groups. Correlation between measures was calculated using Pearson correlation.

## Results

After obtaining 48 CTA from consecutive patients, rendered reconstruction of the LVS was made, and data was collected. Patient characteristics are presented in Table 1. The collected data from the LVS can be divided into three groups: measurements of the arterial branches in the LVS region, measurement and calculation of LVS surface area, and LAA type and coverage over the LVS.

**Table 1:** Patient characteristics, IFG (impaired fasting glycemia)

Characteristic	Value
Baseline	
Age	57.87 ± 11.56
Sex	Male – 20 (41.7%)
BMI	29.17 ± 4.8
Hypertension	*n* 36; 75%
Diabetes mellitus/IFG	*n* 8; 16.7%/*n* 6; 12.5%
Heart failure	*n* 8; 16.7%
Ischemic heart disease	*n* 14; 29.16
Left ventricle ejection fraction	58.8 ± 8.48 (30–70)
History of atrial fibrillation	*n* 2; 4.16%
History of ventricular arrhythmia	*n* 10; 20.83%

### Measurements of the arterial branches in LVS region

**Fig. 1:**
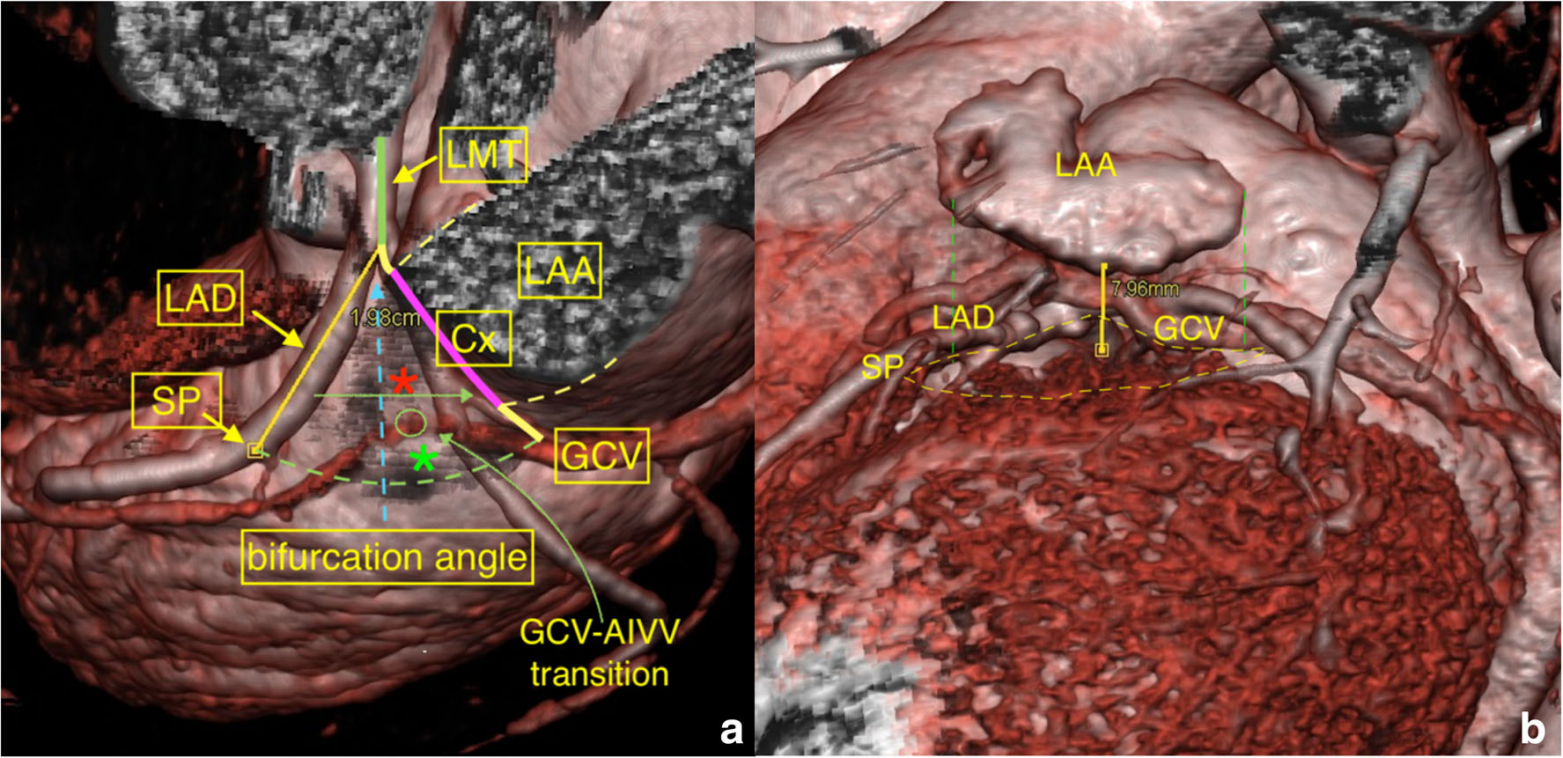
**a** LVS, green line; LMT left main trunk, LAA left atrial appendage, GCV great cardiac vein, SP septal perforator, LAD left anterior descending. Yellow line: Cx from bifurcation to right—proximal margin of LAA. Violet line: Cx from bifurcation to distal—left margin of LAA. Red asterisk: inaccessible area. Green asterisk: accessible area. Green circle over GCV: point of transition GCV–AIVV. **b** Distance from the LAA to the surface of the LVS

The mean length of the LCA (trunk) was 10.54 ± 4.18 mm, distance to first dominant SP was 20.11 ± 4.33 mm, the shortest distance to the first dominant SP was 10 mm, while the most extended was 28 mm. The mean angle found in the LCA bifurcation was 82.21° (± 18.91) (Fig. 1). All measured values are shown in Table 2.

**Table 2:** Measurements of the LVS, clear LVS—with no (RI)—ramus intermedius or branches form LAD or CX

Measurements of the LVS	Value (SD)
Left main trunk	10.54 ± 4.18 (3–21 mm)
Distance LAD—sp (first dominant)	20.11 ± 4.33 (10–28 mm)
Angle of LAD and Cx	82.21 ± 18.91 (48–125 deg)
LVS from formula [F]	291.58 mm^2^ ± 115.5 (91.58–556.04mm^2^)
LVS from ct image [CT]	263.33 mm^2^ ± 99.49 (59.22–447.11)
LVS superior aspect	133.42 ± 72.89
LVS inferior aspect	95.67 ± 72.77 (*n* 7 with no inferior aspect)
LVS formula/LVS CT image	0.91 ± 0.12, 95% CI 0.034, *p* = 0.44
Accessory coronary vessels*	RI, 14; Dx1, 14; Dx2, 3; Mg1, 20; Mg2, 0
CLEAR LVS	*n* 12.25%
Correlation [F] vs [CT]	= 0.93, *p* < 0.0001

#### Measurements of the LVS

**Fig. 2:**
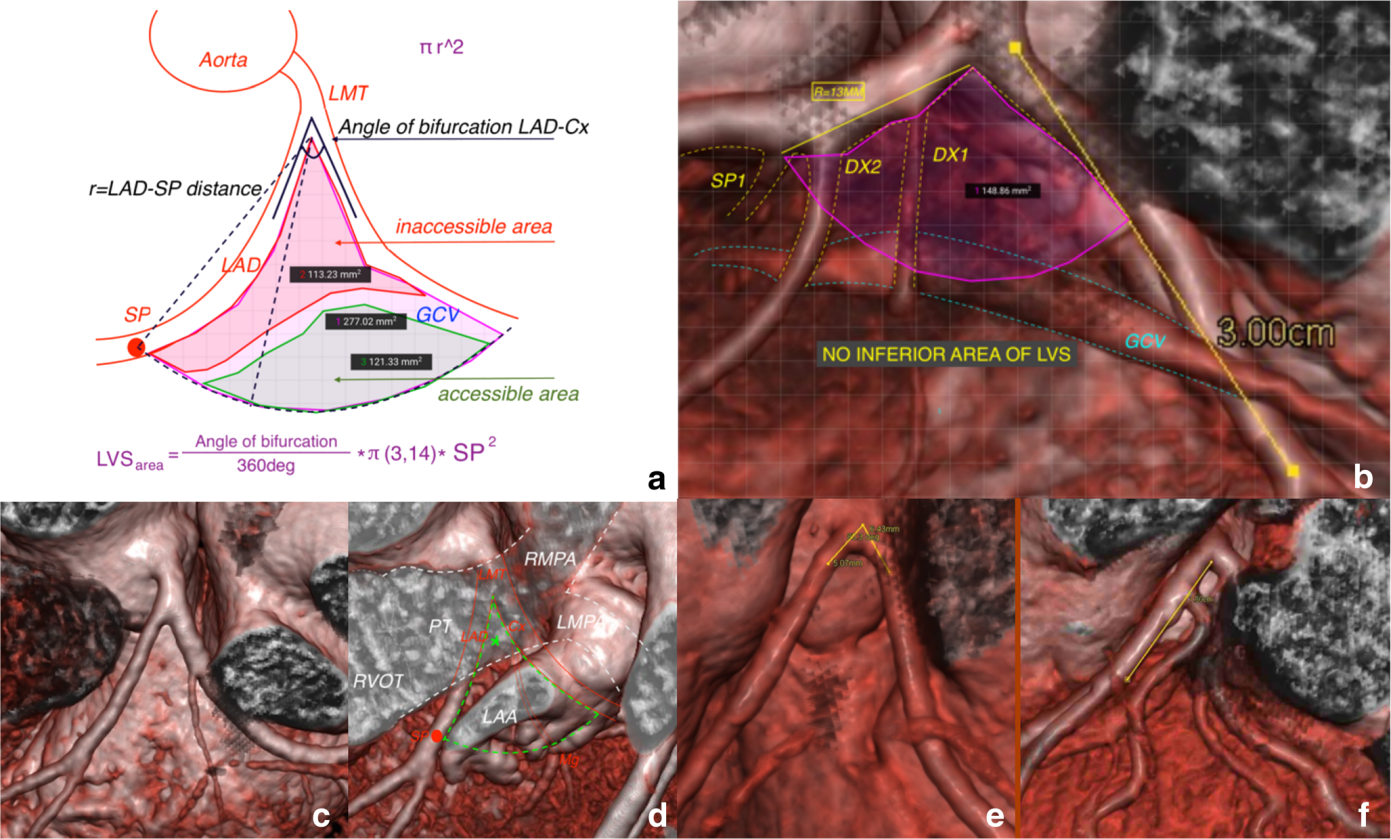
**a** Yamada equation for calculating the LVS area. LMT left main trunk, LAD left anterior descending, SP septal perforator, GCV great cardiac vein. **b** LVS with no accessible area. SP1 first septal perforator, Dx diagonal branches. **c**, **d** Overlap of right ventricular output tract (RVOT) and pulmonary trunk over LVS. PT pulmonary trunk, LMPA left main pulmonary artery, RMPA right main pulmonary artery, LAA left atrial appendage. **e** Clear LVS. **f** LVS with multiple coronary vessels

Guided by the definition of Yamada, we prepared the equation presented in Fig. 2a. The angle at the bifurcation and distance to the SP was used to calculate the area of the LVS. The mean LVS area calculated from the formula was 291.58 mm^2^ (± 115.5) (Fig. 2a), while the measured area from the rendered image in SketchAndCalc software was 263.33 mm^2^ (± 99.49)—9.1%, hence less than the calculated area. The correlation between the LVS area and the formula with the CT image was 0.93, which is a strong positive correlation (*p* < 0.0001). The confidence interval (95% CI) for the difference was 0.034. Also, a Student’s *t* test reveals a *p* = 0.44, indicating no significant difference between the formula and CT scan imaging. The size of the LVS may vary, from the smallest at 59.22 mm^2^ to the largest at almost 7× the size at 447.11 mm^2^. The mean inaccessible area (superior) was 133.42 mm^2^ (± 72.89) and accessible area (inferior) 95.67 mm^2^ (± 72.77). In 7 inspected hearts, there was no accessible area due to the short distance to the first SP and the specific course of the GCV (Fig. 2b). We found a significant difference between the size of the superior and inferior aspect of the LVS (*p* = 0.006). BMI correlates with size of the field of LVS (*p* = 0.01) and accessible area (*p* = 0.01).

The region of the LVS is also very diverse. In Fig. 2c, d, we observe an overlap of the right ventricular outflow tract (RVOT) over the LVS, especially over the inaccessible area. Another worthy point to observe would be in between the RVOT and the LVS, which presents with the LAA passing over the point of transmission of the distal GCV to the AIVV. Figure 2e and f present a diversity of vessels in the LVS. In Fig. 2e, the LVS is free from any branches of the coronary vessels, while in Fig. 2f is covered by many branches from the LAD and Cx. In the presented cases, we found 14 ramus intermedius (RI), 14 first diagonal branches (Dx), 3 s Dx, and 20 first marginal branches (Mg1) from the circumflex coronary artery. Only 12 of the LVS were out of accessory coronary vessels. All collected data are presented in Table 2.

#### LAA types and coverage over LVS

**Fig. 3:**
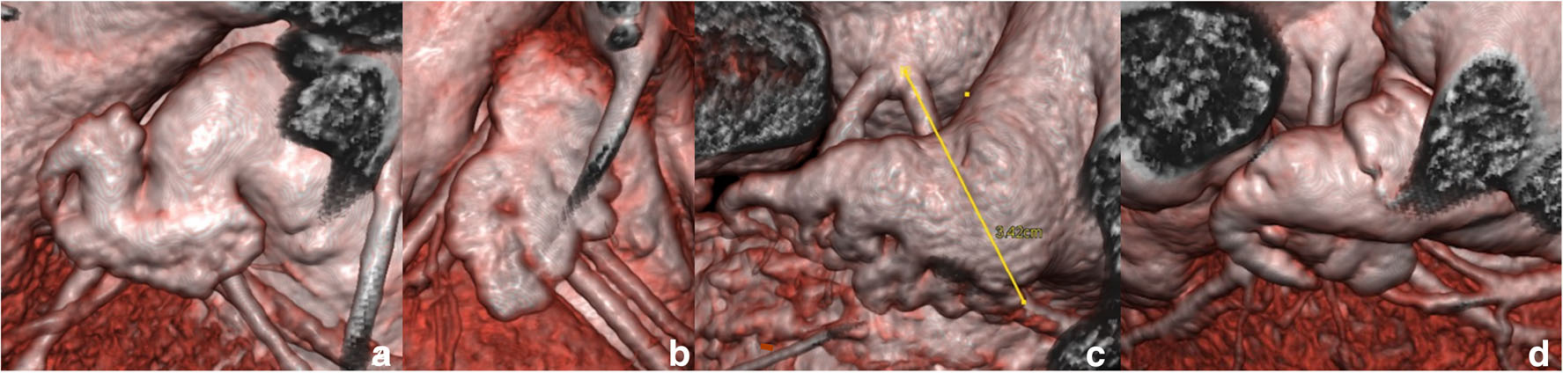
Four types of LAA. **a** Chicken wing. **b** Windsock. **c** Broccoli. **d** Cactus shape

Four main types of the LAA were found and categorized by shape in the study group: Twelve “cactus” (25%), 24 “chicken wing” (50%), 8 “windsock” (16.7%), and 4 “broccoli” (8.3%) (Fig. 3). Distance between the bifurcation of the LAD-Cx to the right margin of the LAA was 3.54 ± 3.58 mm (0–11 mm) and to the left margin of the LAA was 22.99 ± 5.81 mm (12 to 36 mm); overall, the average width over the LVS of the LAA was 19.45 ± 4.78 mm. The average distance between the LAA to the LVS surface was 5.14 ± 2.47 mm (1.4–10 mm) (Fig. 1b). This distance was related to the thickness of the epicardial adipose tissue in this region. At the same time, it was not associated with the shape of the LAA and showed no significant relationship to BMI, age, or gender.

**Fig. 4:**
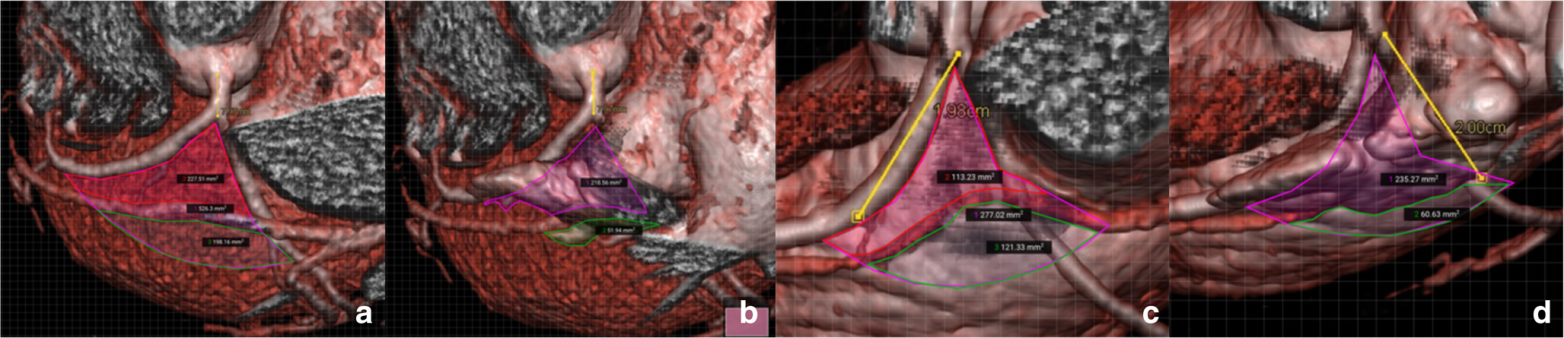
Measurements of LAA coverage over LVS. **a**, **b** LVS without and with LAA. **c**, **d** Different example

The mean LAA coverage over the LVS was 195.01 mm^2^—which is approximately 75% of the LVS area, 75% over the inaccessible area, and 59% over accessible area (Fig. 4). The highest coverage was measured from the broccoli shape at 95.55%, while the smallest coverage was from the cactus at 60.54%. The coverage over the inaccessible area was most extensive from the broccoli shape (93.1%) and less from the cactus shape (46.99%). Above the accessible area, the highest coverage was from the windsock shape (80.23%) and the smallest from the cactus shape (46.99%). All detailed measurements are shown in Table 3.

**Table 3:** Measurements of coverage over the LVS in each type of LAA. The critical point was called a point of transition from the distal part of GCV to the AIVV (green circle in Fig. 1a). Inf = inferior, Sup = superior, Prox = proximal, Dist = distal

LAA	Cactus	Chicken wing	Windsock	Broccoli	All
Number	*n* 12 (25%)	*n* 24 (50%)	*n* 8 (17%)	*n* 4 (8%)	*n* 48 (100%)
LAA prox–dist (mm)	18.97 ± 5.74	19.72 ± 4.88	19.87 ± 4.73	18.5 ± 6.35	19.45 ± 5.05
LAA over LVS (mm^2^)	201.37 ± 138.30 (60.54 ± 25.7%)	179.66 ± 86.72 (75.09 ± 23.5%)	213.93 ± 41.02 (83.31 ± 13.94)	243.01 ± 44.11 (95.55 ± 6.3%)	196.08 ± 94.43 (75.08 ± 24.62%)
LAA over sup (mm^2^) Inaccessible area	121.07 ± 97.34 (62.43 ± 20.41%)	89.83 ± 65.1 (75.50 ± 23.99%)	109.71 ± 30.13 (85.63 ± 19.44%)	146.55 ± 44.19 (93.1 ± 5.67%)	105.68 ± 69.89 (75.39 ± 22.91%)
LAA over inf (mm^2^) accessible area	61.11 ± 91.79 (46.99 ± 41.73%) No cover, 4	66.3 ± 68.2 (59.89 ± 41.45%) No cover, 5	57.82 ± 26.38 (80.23 ± 33.13%) No cover, 0	77.57 ± 89.57 (47.72 ± 55.1%) No cover, 2	64.53 ± 69.93 (59.04 ± 41.6%) No cover, 11
LAA distance to LVS (epicardial) (mm)	5.29 ± 1.79 Min 2.9 Max 8	4.58 ± 2.52 Min 1.5 Max 9.5	5.62 ± 3.1 Min 2 Max 10	7.12 ± 2.01 Min 5 Max 9.5	5.14 ± 2.47 Min 1.5 Max 10
Critical point coverage	*n* 11 (91.67%)	*n* 24 (100%)	*n* 8 (100%)	*n* 4 (100%)	*n* 47 (98%)

## Discussion

For over 20 years, the radiofrequency catheter ablation (RFCA) has been an effective and safe nonpharmacological therapy for arrhythmias. Idiopathic left ventricular outflow tract arrhythmias have varying origins, and successful ablation may be challenging [7, 8]. Four types of LVOT-VA origins are known to exist within very close anatomical proximity. Researchers using a variety of methods, mainly 3D electro-anatomical systems establish four main origins of LVOT-VAs. These are the aortomitral continuity (AMC), the aortic sinus cusps (ASC), the anterior sites surrounding the mitral annulus (MA), and the epicardium [9]. While the first three locations with precise 3D mapping and adequate power are highly accessible for successful ablation, the epicardial origin of the arrhythmia has an anatomical boundary and may require a more problematic approach. Access to the epicardial arrhythmias may be provided through the venous system via the coronary sinus to GCV and AIVV, trans-pericardial approach, or under favorable anatomical circumstances from the RVOT/pulmonary artery (Fig. 2c, d) As a result, during an ablation procedure, the RVOT area and pulmonary artery are usually mapped [9].

Ablation from distal GCV, close to its continuation as the anterior interventricular (AIV), is a highly successful procedure [3], but anatomy of the cardiac venous system may prevent reaching the transition point, even with high-flow saline perfusion, 60 mL/min at a period of 2 min [10]. Percutaneous, pericardial instrumentation for epicardial catheter mapping and ablation of the LVS is feasible, safe, and may be applicable to a variety of cardiac arrhythmias in which standard endocardial ablation techniques have failed [11] unless the operating team is inexperienced in such technique. Omitting all aforementioned challenges, mapping ventricular prepotentials from the LAA via transseptal puncture seems to be comfortable and feasible for moderately experienced electrophysiologists.

The anatomy of the LAA is well revised [12–14]. Internal structures lined by the pectinate muscle bundles in the LAA do not ramify like the teeth of a comb. These diverse surfaces may have an important issue in proximity to the LV surface; however, the wall of the appendage is paper-thin between the muscle bundles [13] and gives an opportunity for undisrupted mapping of prepotentials and ablation from this site.

The main question was to assess the anatomical functionality of the LAA as a mapping area for LVS arrhythmias. We enacted a thesis that measuring the distance from the LMT bifurcation to the first dominant septal perforator, and the angle of bifurcation, will be enough to assess the size of the LVS. The formula (Fig. 2a) against the real size of the LVS was comparable with a size difference of less than 10%, with no statistical significance (*p* = 0.44) with a strong positive correlation (= 0.93, *p* < 0,0001), and can be used interchangeably. The LAA coverage is over 75% of the LVS, and 98% of it lies on the critical point—GCV-AIVV. The best coverage was found in the “broccoli” shape at 95%; however, it was the less common LAA type. It appears that the “windsock” type, because of length and monomorphic structure, has the most perspective for arrhythmia mapping. Presence of coronary branches in the LVS was not uncommon. The superior area is also named the inaccessible area for the epicardial approach to catheter ablation because of the close proximity of the coronary arteries and the thick layer of epicardial fat that overlies the proximal portion of these vessels [2].

Nevertheless, can we call the inferior LVS as always accessible? According to the definition, 14.6% of the hearts did not have any inferior aspect of the LVS, while only 25% of all hearts had a clear LVS. Without CA before the procedure, mapping or ablating the LVS region may be hazardous, and it is recommended [15]. CA visualizes all branches from the left coronary artery, the first septal perforator, and angle of bifurcation for measurement of the LVS. Acquiring a potential map of the LAA using CA or CTA allows us to choose the best procedural choice for the patient and prevent unnecessary ablation in suboptimal regions.

The last issue is the distance from the LAA to the LVS, which may be an insurmountable barrier. Data showed that the distance between the two structures might vary from 1.4 to 10 mm. In some cases, it is enough even for nonirrigated electrodes. The depth of the lesions created by a RF pulse is similar to the thickness of the left atrium (3 to 5 mm) and such an application is unlikely to reach intramural or endocardial circuits [4]. Using the open irrigated RF electrode, the depth is greater than 7 mm [16], with contact force electrode the effective power delivery to the underlying tissue is significantly higher [17]. Using a retractable needle-tipped catheter might be too dangerous causing cardiac tamponade [18], but using a bipolar approach can be a new frontier in this approach [19].

Kumgai [9] reported that all subtypes of LVOT-VAs, except those with epicardial origins, are successfully treated with endocardial radiofrequency catheter ablation combined with pace mapping and the identification of the earliest ventricular electrogram with a prepotential, if it is recordable. Currently, the LAA is opening a new frontier for LVS epicardial ablations using the endocardial approach.
